# ESIPT-Based Photoactivatable Fluorescent Probe for Ratiometric Spatiotemporal Bioimaging

**DOI:** 10.3390/s16101684

**Published:** 2016-10-12

**Authors:** Xiaohong Zhou, Yuren Jiang, Xiongjie Zhao, Dong Guo

**Affiliations:** 1 College of Chemistry and Chemical Engineering, Central South University, Changsha 410083, China; zhouxh001@csu.edu.cn (X.Z.); 152301025@csu.edu.cn (X.Z.); guo4675@csu.edu.cn (D.G.); 2 Environment Monitoring Department, Changsha Environmental Protection College, Changsha 410004, China

**Keywords:** ESIPT, photoactivatable, ratiometric

## Abstract

Photoactivatable fluorophores have become an important technique for the high spatiotemporal resolution of biological imaging. Here, we developed a novel photoactivatable probe (PHBT), which is based on 2-(2-hydroxyphenyl)benzothiazole (HBT), a small organic fluorophore known for its classic luminescence mechanism through excited-state intramolecular proton transfer (ESIPT) with the keto form and the enol form. After photocleavage, PHBT released a ratiometric fluorophore HBT, which showed dual emission bands with more than 73-fold fluorescence enhancement at 512 nm in buffer and more than 69-fold enhancement at 452 nm in bovine serum. The probe displayed a high ratiometric imaging resolution and is believed to have a wide application in biological imaging.

## 1. Introduction

Photoactivatable small-molecule fluorescent probes have become an indispensable tool for spatiotemporal microscopic studies of tissues and animals [1,2,3]. In the past few years, several photoactivatable probes generated from fluorescent proteins (FPs) [4,5,6,7,8,9], quantum dots [10,11] or organic fluorophores [12,13] have been widely developed for high-quality bioimaging with temporal and spatial resolution. Among them, fluorescein [14], coumarin [15], rhodamine [16], FRET-based dyes [17], and so on, have attracted particular attention based on their excellent photophysical features. For an organic molecular photoactivatable probe, a caging group is usually adopted to protect the fluorophore and quench the fluorescence [18,19,20,21,22,23]. Under experimental conditions, the photosensitive bond is photocleaved by ultraviolet light (UV), resulting in the release of the caging group and recovery of fluorophore fluorescence. Thus, it is theoretically possible to endow these probes with high spatiotemporal resolution by controlling both the illumination time and site. Unfortunately, upon UV stimulation, almost all primarily reported photoactivatable organic probes have been based on single emission intensity change, which tends to be affected by various environmental factors, hence reducing spatial resolution. Ratiometric probes can eliminate these interferences by the built-in correction of the dual emission bands, resulting in a more favorable system for imaging living cells.

2-(2-Hydroxyphenyl)benzothiazole (HBT) is a small organic dye, known for its classic luminescence mechanism through ESIPT [24,25,26,27,28,29]. Importantly, HBT has high quantum yield with highly desirable photophysical properties, such as intense luminescence, a large Stokes shift (>130 nm) and significant photostability [30,31]. By replacing the phenolic hydrogen of HBT with a photocaged group, the fluorescent HBT becomes lightless by blocking ESIPT, because the molecule cannot form the fluorescent structure (enol and keto forms) with dual emission. These unique features make HBT different from common fluorescent dyes and, thus, favorable for the design of activatable probes that release a dual emission fluorophore with a localizable fluorescence signal and high spatial resolution.

**Scheme 1:**
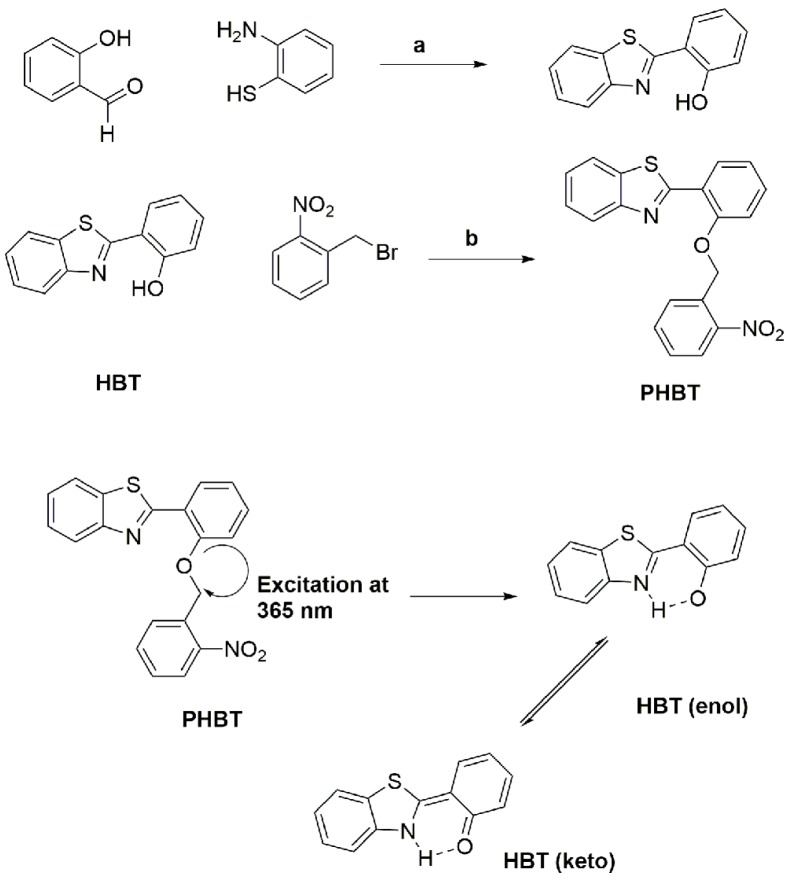
Synthetic route for the photoactivatable fluorescent probe PHBT and the response mechanism. (**a**) R: H_2_O_2_ (30%), R: (NH_4_)_2_Ce(NO_3_)_6_, S: MeCN, 30 min, room temperature; (**b**) R: K_2_CO_3_, S: MeCN, 24 h, 70 °C.

Accordingly, quite a few HBT-based probes have been developed over the past two decades [32,33,34,35,36,37]. When the protecting group is removed, a bright fluorescent ratiometric product is released from these probes at the site of analytes’ reaction. This effectively solves the single-emission problem faced by common probes and enable good spatial resolution in cells. Inspired by these successful designs, we reported herein the use of HBT as a precursor to develop a photoactivatable probe (PHBT) for spatiotemporal bioimaging applications (Scheme 1). PHBT contains a 2-nitrobenzyl group that serves as both a photosensitive moiety and a fluorescence quenched unit. When triggered with UV light, the probe released an ESIPT fluorophore-HBT that showed strong yellow-green fluorescence in aqueous media with more than 73-fold fluorescence enhancement, thereby producing a ratiometric fluorescence signal that affords high spatial resolution for bioimaging. The PHBT was then applied in phototriggered spatiotemporal imaging in live cells, and satisfactory results were obtained.

## 2. Experimental Section

### 2.1. Reagents and Apparatus

All chemicals were obtained from commercial suppliers and without any further purification. Water used in all experiments was doubly distilled. Liquid chromatography-mass spectrometry (LC-MS) analyses were performed using the Agilent 6120 Quadrupole LC/MS from Agilent Technologies. The pH was measured using a pH meter (pH_S_-3C, Shanghai Leici). ^1^H-NMR and ^13^C-NMR spectra were recorded on a Bruker DRX-500 spectrometer operating at 500 MHz. All chemical shifts are reported in the standard notation of parts per million. All fluorescence measurements were carried out on a Hitachi-F4600 fluorescence spectrometer with both excitation and emission bandwidths set at 5.0 nm. Fluorescence images of MDA-MB-231 cells were obtained using a Nikon Te-2500 microscope (Japan).

### 2.2. Spectroscopic Materials and Methods

Stock solution of probe PHBT (1 mM) was prepared by dissolving probe PHBT in dimethyl sulfoxide (DMSO). The test solution of probe PHBT was prepared by placing 10 µL probe PHBT stock solution in 990 µL phosphate-buffered saline (PBS) solution (10 mM, pH = 7.4). Experiments to measure the fluorescence of PHBT were conducted in (10 mmol PBS, pH = 7.4) water/DMSO (99/1, *v*/*v*) solution. Unless otherwise stated, the excitation wavelength was 365 nm; the excitation slit width was 5 nm; and the emission slit width was 5 nm for all measurements.

### 2.3. Probe Synthesis

The Synthetic route to HBT and PHBT is depicted in Scheme 1. A detailed description of the synthesis can be found in the Supporting Information (Figure S1). The product was characterized by ^1^H-NMR, ^13^C-NMR and ESI mass spectra (Figures S2–S4). Compound PHBT: ^1^H-NMR (500 MHz, DMSO) δ = 8.471–8.447 (dd, 1H), 8.227–8.206 (d, 1H), 8.063 (m, 2H), 7.868–7.826 (m, 2H), 7.681 (t, 1H), 7.535 (t, 2H), 7.420–7.418 (m, 2H), 7.369–7.205 (t, 1H), 5.797 (s, 2H); ^13^C-NMR (500 MHz, DMSO) δ = 162.474, 156.037, 152.041, 148.042, 135.756, 134.754, 132.888, 132.189, 130.391, 130.072, 129.603, 126.789, 125.657, 125.484, 122.970, 122.224, 122.186, 121.914, 114.218, 68.108; MS (ESI-MS): *m*/*z* 100% Calcd. for C_20_H_15_N_2_O_2_S^+^ [(M + H)^1+^] 363.1; found 363.1.

### 2.4. Cytotoxicity Test

Cell viability was evaluated by the reduction of MTT (3-(4,5-dimethyl-2-thiazolyl)-2,5-diphenyltetrazolium bromide) to formazan crystals by mitochondrial dehydrogenases (Mosmanm, 1983) (Figure S5). A sample of 1 × 10^5^ MDA-MB-231 cells in 50 μL of washing buffer was seeded into each test well on a 96-well plate. After an overnight culture, PHBT (0–10 μmol/L) in 50 μL of washing buffer was added to the respective test wells. After 2 h of treatment, 10 μL of MTT solution (5 mg/mL in phosphate-buffered solution) were added to each well. After 4 h of incubation at 37 °C, 100 μL of a solution containing 10% sodium dodecyl sulfate (SDS) and 0.01 mol HCl were added to dissolve the purple crystals. After 12 h of incubation, the optical density readings at 595 nm were recorded using a plate reader. Each of the experiments was performed at least three times.

### 2.5. Bovine Serum Test

The test solution of probe PHBT (10 μM) in bovine serum was prepared by placing 10 μL probe PHBT stock solution in 790 μL PBS buffer (10 mM, pH = 7.4) and 200 μL bovine serum. The resulting solution was incubated for 30 min at 37 °C and irradiated under UV light for 30 min before the recording of the spectrum.

### 2.6. Preparation and Staining of Cell Cultures

Immediately prior to the imaging experiments, the MDA-MB-231 cells were washed with PBS, incubated with 10 μM PHBT for 30 min at 37 °C, washed with PBS three times, irradiated by UV 30 min and then imaged. Fluorescence imaging was observed under a Nikon Te-2500 microscope. The laser excitation wavelength was 405 nm, and emissions were centered at 415–460 nm and 470–550 nm.

## 3. Results and Discussion

### 3.1. Design and Synthesis of PHBT

Inspired by the dual-emission optical properties of HBT, a photocaged PHBT was then designed and synthesized. PHBT contains a 2-nitrobenzyl group as a photocaged moiety, as shown in Scheme 1. Upon irradiation with UV light, the 2-nitrobenzyl group of PHBT was cleaved from the probe molecule to release an ESIPT HBT, affording a ratiometric fluorescence signal for imaging in vivo.

### 3.2. Photoactivatable Response Property of PHBT

**Figure 1:**
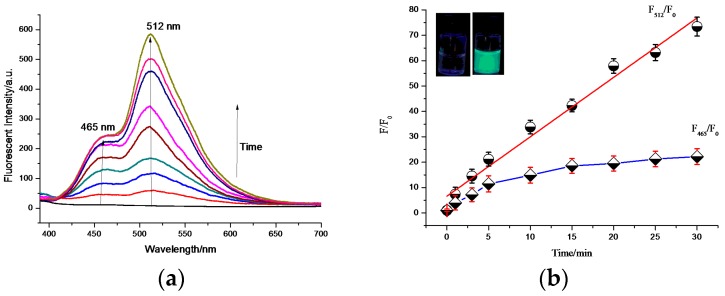
(**a**,**b**) Time-dependent fluorescent spectral changes of PHBT under UV irradiation (0–30 min) at room temperature, PHBT dissolved in PBS buffer (10 mM, pH 7.4, water/DMSO = 99:1) to form 10 μM testing solution, with excitation λ = 365 nm. (inset) Change in the fluorescence of the probe (**left**) before and (**right**) after UV irradiation (30 min) at room temperature.

As shown in Figure 1, the dual emission bands of PHBT were observed under UV light irradiation. The enol tautomer gave emission at 465 nm, and the keto tautomer gave emission at 512 nm. After 30 min, a clear green color could be observed by the naked eyes under a UV lamp (Figure 1). Measurement of the intensity showed a 73-fold enhancement at 512 nm, indicating that PHBT had a good photoactivatable response within 30 min. The quantum yield for the caged probe PHBT was calculated to be 0.0019, and the quantum yield for the uncaged PHBT was 0.17, corresponding to an 89-fold enhancement after photoconversion. Both the photolysis of PHBT and the liberation of HBT were confirmed by the HPLC and UV absorption spectra (Figures S6 and S7).

### 3.3. Bovine Serum Test

**Figure 2:**
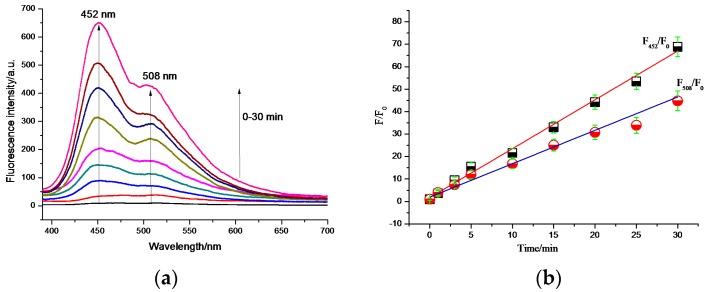
(**a**,**b**) Time-dependent fluorescent spectral changes of PHBT under UV irradiation (0–30 min) at room temperature, PHBT dissolved in PBS buffer (10 mM, pH 7.4, aqueous 20% bovine serum solution) to form 10 μM testing solution with excitation λ = 365 nm.

To test the feasibility of the practical application of the probe PHBT, we further conducted the UV light detection in 20% bovine serum (Figure 2). With irradiation under UV light, the solution of PHBT in bovine serum showed dual emission, as well. However, the fluorescence emission was stronger at 452 nm than that at 508 nm, which is because the forms of the enol tautomer and the keto tautomer are sensitive to the solvent [38,39,40,41,42]. Moreover the interaction between HBT and serum protein can cause the enol form to be much more pronounced [43]. Measurement of the intensity showed a 69-fold enhancement at 452 nm. As shown in Figure 2b, an excellent linear relationship was obtained both at 452 nm and 508 nm.

The fluorescence intensity increased linearly with the time of UV irradiation from 0 min to 30 min, which confirmed that the probe PHBT was applicable for practical phototriggered spatiotemporal imaging in real samples with satisfactory results.

### 3.4. Cell Viability and Imaging in Living MDA-MB-231 Cells

**Figure 3:**
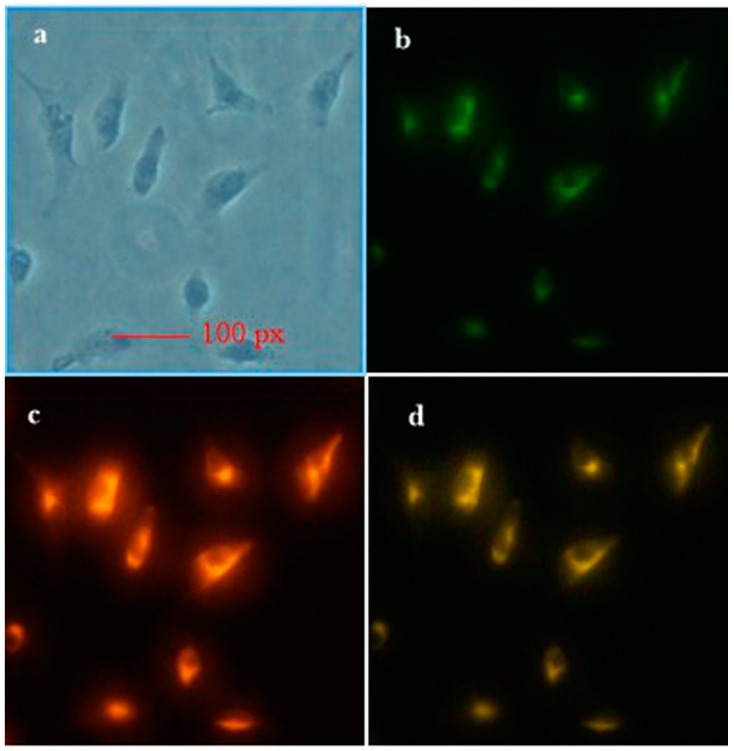
Fluorescent images. MDA-MB-231 cells stained with 10 µM PHBT. (**a**) Bright field image of PHBT in cells; (**b**) green fluorescence of PHBT in cells after 30 min of irradiation at 405 nm; (**c**) red fluorescence of PHBT in cells; and (**d**) overlay of (**b**,**c**).

Previous results demonstrated that PHBT had good chemical and spectral performances. The cytotoxicity of PHBT, HBT and UV irradiation at 365 nm (0–30 min) was then evaluated using an MTT assay (Figure S5). Experimental results showed that negligible cytotoxicity was observed when up to 10 μM PHBT or HBT were added to the culture medium. Moreover, the cytotoxicity of UV irradiation of less than 10 min is also negligible; when the UV irradiation time was more than 10 min, an increased cytotoxicity was observed. The effect of some relevant ions and neutral molecules on the response of the PHBT probe was also investigated. The results are shown in Figure S8; 100 μM of common biologically-relevant species, such as K^+^, Na^+^, Ca^2+^, ClO^−^, H_2_O_2_, vitamin C and cysteine, exhibited a negligible effect on the response of PHBT. In addition, the effect of pH on the fluorescence response of PHBT was also studied, with results added in Figure S9. It is obvious that the probe could achieve the best performance at neutral pH, which is favorable for its applications. MDA-MB-231 cells were incubated with PHBT (10 μM), irradiated by UV 30 min and then imaged with a 405 nm laser line. As shown in Figure 3, the irradiated cell grew bright after irradiation.

## 4. Conclusions

In summary, a novel photocaged fluorescent probe, termed PHBT, for biological studies not only in living cells, but also in bovine serum, has been designed and synthesized. PHBT is based on a small organic fluorophore known for its classic luminescence mechanism through ESIPT with the keto form and the enol form, showing dual emission bands for ratiometric imaging, and the ratiometric signal changes with the deprotection of the photocaged group in the presence of UV light. The experiments demonstrate that PHBT possesses a high ratiometric imaging resolution both in cell and bovine serum and is believed to have a wide application in biological imaging.

## Supplementary Materials

The supplementary materials are available online at http://www.mdpi.com/1424-8220/16/10/1684/s1.

## Abbreviations

PHBT	photoactivatable probe
HBT	2-(2-hydroxyphenyl)benzothiazole
ESIPT	excited-state intramolecular proton transfer
FPs	fluorescent proteins
FRET	fluorescence resonance energy transfer
UV	ultraviolet light
LC-MS	liquid chromatography-mass spectrometry
DMSO	dimethyl sulfoxide
PBS	phosphate-buffered saline
MTT	3-(4,5-dimethyl-2-thiazolyl)-2,5-diphenyltetrazolium bromide
SDS	sodium dodecyl sulfate
