# Supplementary material for: ESIPT-Based Photoactivatable Fluorescent Probe for Ratiometric Spatiotemporal Bioimaging
Source: Sensors (Basel). 2016 Oct 12;16(10):1684. doi: 10.3390/s16101684 (PMC5087472; doi:10.3390/s16101684)
Supplement: Supplementary file 1 [file sensors-16-01684-s001.pdf]

Supplementary Materials: ESIPT-Based Photoactivatable Fluoroscent Probe for Ratiometric Spatiotemporal Bioimaging

Xiaohong Zhou, Yuren Jiang, Xiongjie Zhao and Dong Guo

Calculated the Quantum Yields

The quantum yield of PHBT and HBT under DMSO/H_2O = 1:99 (*v*/*v*)solution (pH 7.4) were calculated by employing rhodamine B (Φ_R = 0.95 in ethanol) as a reference using the following equation: ($\Phi_F = I/I_R \times (n/n_R)^2 \times A_R/A \times \Phi_R$, where Φ_F is the quantum yield, I is the integrated area under the fluorescence spectra, A is the absorbance, n is the refractive index of the solvent and R refers to the reference fluorophore rhodamine B.

Synthesis of Probes

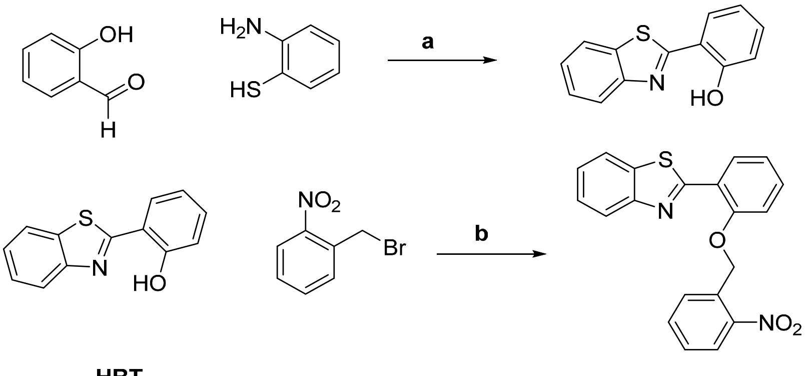

Figure S1. Synthetic route for the Photoactivatable fluorescent probe PHBT. (**a**) R: H_2O_2 (30%), R: $(NH_4)_2Ce(NO_3)_6$, S: MeCN, 30 min, room temperature; (**b**) R: K_2CO_3, S: MeCN, 24 h, 70 °C.

(a) Synthesis of HBT[1]

In a round-bottomed flask (50 mL) equipped with a magnetic stirrer, a solution of 1,2-phenylenediamine (125 mg, 1 mmol), salicylic aldehyde (122 mg, 1 mmol) in MeCN (3 mL) was prepared. H_2O_2 (30%, 0.4 mL, 4 mmol,) and $(NH_4)_2Ce(NO_2)_6$ (54.8 mg, 0.1 mmol) were added, and the mixture was stirred at room temperature for 30 min. Then, the reaction mixture was quenched by adding water (10 mL), extracted with ethyl acetate (4 × 10 mL) and dried with anhydrous $MgSO_4$. The filtrate was evaporated and the only product HBT (204 mg, 0.9 mmol) was obtained.

(b) Synthesis of PHBT[2]

A 227 mg (1 mmol) sample of 2-(2-hydroxyphenyl)benzothiazole was added to a 50 mL flask with a reflux condenser. At the same time, 216 mg 2-nitrobenzyl bromide (1 mmol), 276 mg (2 mmol) K_2CO_3 and 10 mL of acetonitrile were added, and the mixture was stirred at 70 °C overnight under protection from light. Then, the mixture was filtered and the solvent was evaporated by rotary vacuum, followed by fast column chromatography (petroleum ether/ethyl acetate = 5/1, *v*/*v*) to obtain 308 mg (0.85 mmol) PHBT.

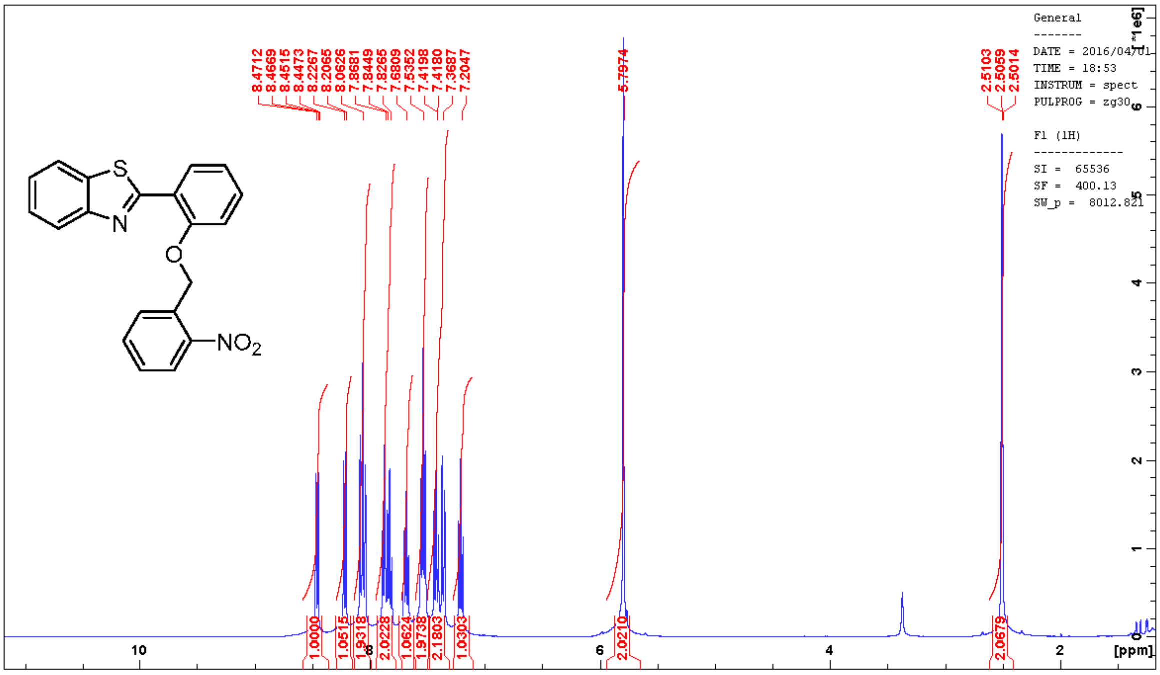

Figure S2. ^{1}H-NMR of PHBT in DMSO-d_6.

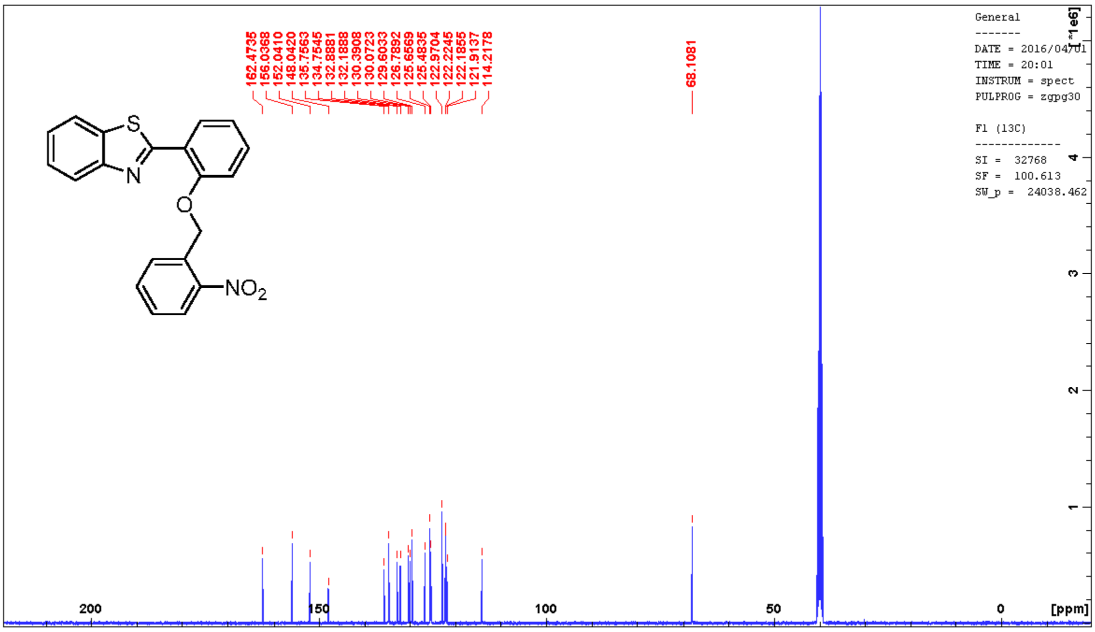

Figure S3. ^{13}C-NMR of PHBT in DMSO-d_6

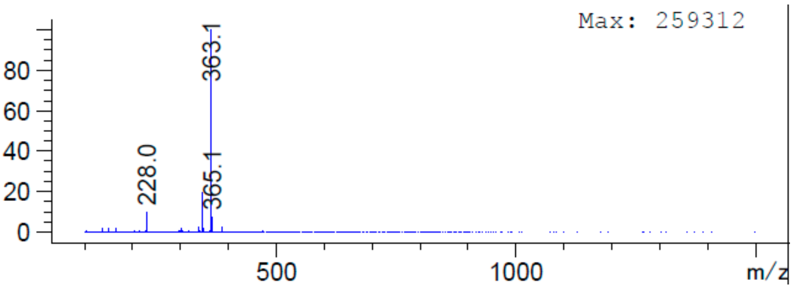

Figure S4. ESI-MS spectra of PHBT.

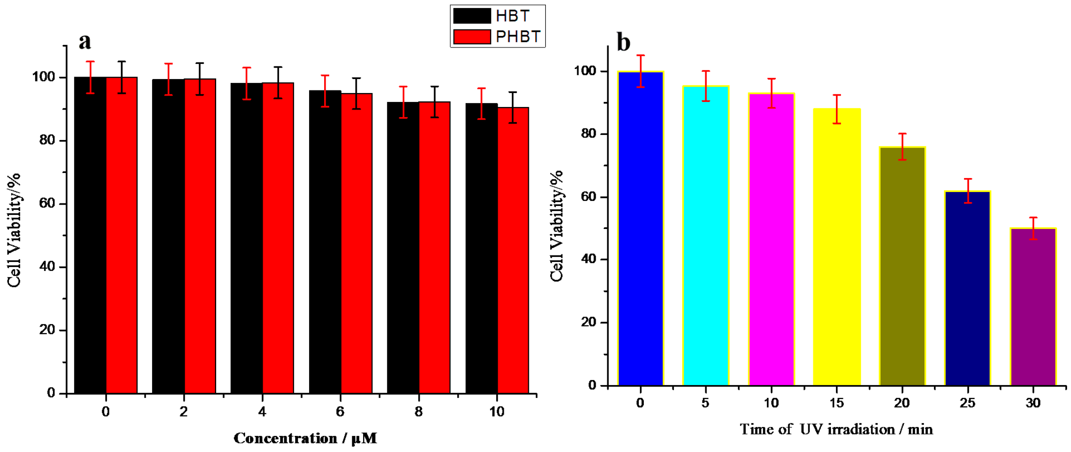

Figure S5. (**a**) Cytotoxicity of PHBT and HBT (0 ~ 10 µM) against MDA-MB-231 cells, as determined by the MTT assay; (**b**) cytotoxicity of PHBT (10 µM) after UV irradiation at 365 nm (0 ~ 30 min) against MDA-MB-231 cells, as determined by the MTT assay.

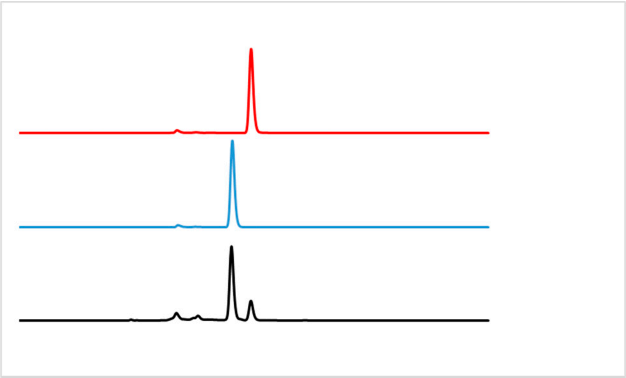

Figure S6. HPLC spectra of PHBT (10 µM, **blue** line), HBT (10 µM, **red** line) and PHBT (10 µM, **black** line) after irradiation by UV light at 365 nm for 30 min in PBS-buffer (DMSO 1%) at pH 7.4.

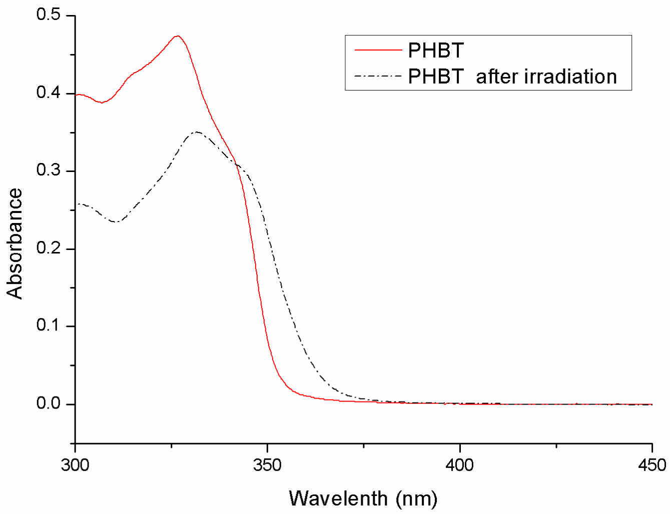

Figure S7. Absorption spectra of PHBT (10 µM) (**red** line) and PHBT (10 µM) (**black** line) after irradiation by UV light at 365 nm for 30 min in PBS-buffer (DMSO 1%) at pH 7.4.

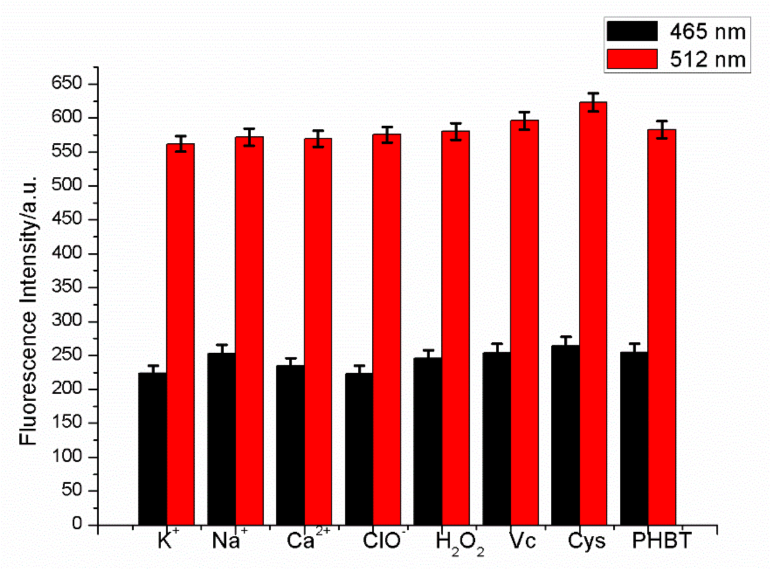

Figure S8. Fluorescence response of probe PHBT (10 μM) to some biologically relevant species (100 μM): K^+; Na^+; Ca^{2+}; ClO^-; H_2O_2; vitamin C and cysteine after UV radiation for 30 min.

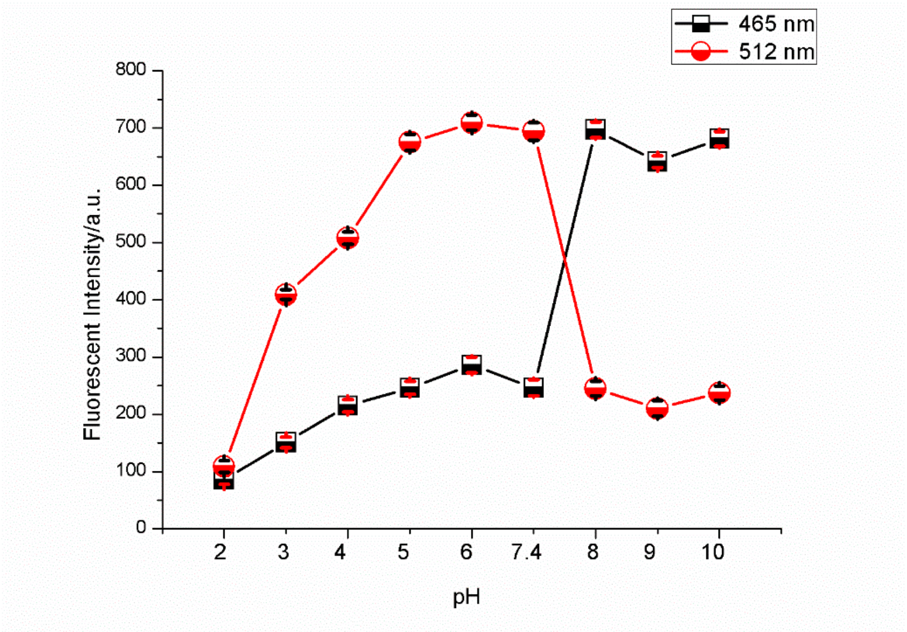

Figure S9. Effect of pH on the fluorescence intensity of probe PHBT (10 μM) after UV radiation for 30 min in buffer (pH 2.0–10.0, 10 mM), the pH values were adjusted by an aqueous solution of NaOH (aq, 1 mM) or HCl (aq, 1 mM), with excitation λ = 365 nm.

References

1. Bahrami, K.; Khodaei, M.M.; Naali, F. ChemInform Abstract: Mild and Highly Efficient Method for the Synthesis of 2-Arylbenzimidazoles and 2-Arylbenzothiazoles. *J. Org. Chem.* **2008**, *73*, 6835–6837.
2. Carvalho, J.F.S.; Louvel, J.; Doornbos, M.L.J.; Klaasse, E.; Yu, Z.Y.; Brussee, J.; Ijzerman, A.P. Strategies to reduce HERG K+ channel blockade. Exploring heteroaromaticity and rigidity in novel pyridine analogues of dofetilide. *J. Med. Chem.* **2013**, *56*, 2828-2840.
